# Response to: Cost effectiveness of intracameral cefuroxime prophylaxis and its efficacy in preventing endophthalmitis after cataract surgery at a referral hospital

**DOI:** 10.5935/0004-2749.2023-0245

**Published:** 2023-11-24

**Authors:** Goksu Alacamli

**Affiliations:** 1 Ophthalmology Departmant, Trakya University Faculty of Medicine and Education and Research Hospital, Edirne, Turkey

Dear Editor,

We have read with great interest the article by Lívia da Silva Conci, Arthur Pinheiro Favarato, and Alexandre Grobberio Pinheiro^(1)^. In response to this article^(1)^, which we assert is a well-thought out and written paper, I am wondering if blepharitis was preoperationally diagnosed for patients who were diagnosed with endophthalmitis after the operation. As we already know and based on the literature, blepharitis is a known risk factor for endophthalmitis^(2,3,4)^. At our clinic, the Trakya University Faculty of Medicine and Education and Research Hospital, we applied the same dose of cefuroxime at the end of the operation intracamarally, for approximately 25 years. Our faculty of medicine hospital, which is a tertiary referral hospital, has a low rate of endophthalmitis. As described in a past article^(1)^, this type of usage of cefuroxime is extremely cost effective in the preventive treatment of endophthalmitis. However, despite the preventional therapies such as the application of 5% povidon iodine before the cataract surgery and cefuroxime injection at the end of the operation, in patient depented insufficiencies, such as diabetes mellitus, blefaritis, and immunsufficiencies, endophthalmitis can occur. I wonder whether there exists preoperational data on the clinical features of patients diagnosed with endophtalmitis after the operation in the present study^(1)^. In the article by Conci et al.^(1)^, efficiency of cefuroxime injection is well described. We are indeed indebted to all the authors^(1)^.

Despite the advances made in the treatment and diagnosis of endophthalmitis, postoperative endophthalmitis (POE) still cannot be entirely managed after a cataract surgery^(5)^. The complete pathophysiology of endophthalmitis also remains to be clarified^(5)^. Thus, to reduce the overall risk, all preventinal actions must be considered.
