# Supplementary material for: Response to: Cost effectiveness of intracameral cefuroxime prophylaxis and its efficacy in preventing endophthalmitis after cataract surgery at a referral hospital
Source: Arq Bras Oftalmol. 2023 Nov 24;87(1):e2023-0245. doi: 10.5935/0004-2749.2023-0245 (PMC11587502; doi:10.5935/0004-2749.2023-0245)
Supplement: Related texts

# Reply: Response to Letters to Editor: Cost effectiveness of intracameral cefuroxime prophylaxis and its efficacy in preventing endophthalmitis after cataract surgery at a referral hospital

**Authors:** Lívia da Silva Conci, Arthur Pinheiro Favarato, Alexandre Grobberio Pinheiro

**Affiliations:** 1 Department of Ophthalmology, Universidade Federal do Espírito Santo, Vitória, ES, Brazil; 2 Department of Ophthalmology, Universidade Estadual de Campinas, Campinas, SP, Brazil

Dear Editor,

We thank you for the opportunity to respond to Dr. Alacamli’s letter and clarify the issues regarding our study. We also extend our gratitude to Dr. Alacamli for taking an interest in our publication and expressing his concerns.

The aim of our study was to evaluate the clinical and economic impact of intracameral cefuroxime administration so as to prevent endophthalmitis occuring during a cataract surgery^(1)^. Our results indicated that the incidence of endophthalmitis decreased by 86% after introducing intracameral cefuroxime prophylaxis at the study hospital^(1)^. In addition, our approach had an economic impact of the numbers of patients who needed the treatment, which was 568^(1)^. The potential savings with cefuroxime was thus approximately US $2,334.36 for every 568 patients who were treated^(1)^. Therefore, we conclude that intracameral cefuroxime intervention is an effective and cost-effective approach toward preventing endophthalmitis after phacoemulsification surgeries^(1)^.

However, notably, our study has several limitations owing to its retrospective nature. Unfortunally, we could not obtain the exact clinical details on endophthalmitis’ risk factors, such as preoperative blepharitis. This data could have added further relevanance to our study findings. Despite these limitations, our study corroborates the clinical trial results in a large clinical practice outcomes database, which further confirms the value of prophylactic cefuroxime administration.

Once again, we deeply thank Dr. Alacamli for his contribution to this discussion.

## References

[1] Conci LD, Favarato AP, Pinheiro AG. (2023). Cost effectiveness of intracameral cefuroxime prophylaxis and
its efficacy in preventing endophthalmitis after cataract surgery in a
referral hospital. Arq Bras Oftalmol [Internet].
